# High-sensitivity Troponin T in hemodialysis patients: a randomized placebo-controlled sub-study investigating angiotensin-II-blockade, variation over time and associations with clinical outcome

**DOI:** 10.1186/s12882-020-02103-1

**Published:** 2020-10-28

**Authors:** Christian D. Peters, Krista D. Kjaergaard, Kent L. Christensen, Bo M. Bibby, Bente Jespersen, Jens D. Jensen

**Affiliations:** 1 Department of Renal Medicine, Aarhus University Hospital, Palle Juul-Jensens Boulevard 99, 8200 Aarhus N, Denmark; 2 Department of Clinical Medicine, Aarhus University, Aarhus, Denmark; 3 Department of Cardiology, Aarhus University Hospital, Aarhus, Denmark; 4 Department of Biostatistics, Aarhus University, Aarhus, Denmark

**Keywords:** Angiotensin II blockade, Troponin T, Hemodialysis, Randomized controlled trial, Variation, Irbesartan

## Abstract

**Background:**

Troponin T (TnT) is a well-known risk factor for negative outcome in hemodialysis (HD) patients, but little is known about variation over time, and the impact of clinical and dialysis specific factors. This study investigated the effect of angiotensin II receptor blockade (ARB), short and long-term variation in TnT and associations with clinical parameters.

**Methods:**

In this analysis based on the SAFIR-cohort (Clinical Trials ID: NCT00791830) 81 HD patients were randomized double-blind for placebo (*n* = 40) or angiotensin II receptor blocker (ARB) treatment (*n* = 41) with irbesartan (150–300 mg) and followed for 12 months with six serial measurements of TnT using a high-sensitivity assay.

**Results:**

Fifty-four patients (67%) completed follow-up. Baseline TnT-medians (min-max) were (placebo/ARB): 45(14–295)/46(10–343) ng/L. ARB-treatment did not significantly affect mean TnT-levels over the 12-month study period. Median week-to-week and one-year TnT-variation (5th–95th-percentile range) using all samples regardless of intervention were: 0(− 14–10) ng/L (week-to-week) and 3(− 40–71) ng/L (12 months). Median TnT-amplitude, capturing the change from the lowest to the highest TnT-value observed during the one-year study period was 38% or 20.5 ng/L. Median ratios with 95% limits of agreement were: 1.00(0.73–1.37); *P* = 0.92 (1 week/baseline; *n* = 77) and 1.07(0.52–2.25); *P* = 0.19 (12 months/baseline; *n* = 54). Baseline TnT was positively correlated with diabetes, ultrafiltration volume, arterial stiffness, change in intradialytic total peripheral resistance and N-terminal pro b-type natriuretic peptide (NT-proBNP) and negatively correlated with hematocrit, residual renal function and change in intradialytic cardiac output. High baseline TnT was associated with a higher risk of admission and cardiovascular (CV) events during follow-up. Increase in TnT over time (ΔTnT = 12-months-baseline) was significantly associated with increase in left ventricular (LV) mass and NT-proBNP and decrease in LV ejection fraction and late intradialytic stroke volume. ΔTnT was not significantly associated with admissions, CV or intradialytic hypotensive events during follow-up. Admissions were significantly more likely with a high (TnT-amplitude> 20.5 ng/L) than a low TnT-amplitude. Peaks in TnT were less frequent in aspirin-treated patients.

**Conclusion:**

ARB-treatment had no significant effect on TnT-levels. Week-to-week variation was generally low, yet over 12 months individual patients had considerable TnT fluctuations. Rise in TnT over time was significantly correlated with markers of cardiac deterioration.

**Trial registration:**

ClinicalTrials.gov Identifier: NCT00791830. Date of registration: November 17, 2008. EudraCT no: 2008–001267-11.

**Supplementary information:**

**Supplementary information** accompanies this paper at 10.1186/s12882-020-02103-1.

## Background

Hemodialysis (HD) patients have a high prevalence of cardiovascular (CV) disease and increased risk of myocardial infarction (MI) [1]. Troponin T (TnT) is a small protein (37 kDa), which acts as the tropomyosin-binding and thin filament-anchoring subunit of the troponin complex, that regulates contraction in cardiac and skeletal muscles [2]. The development of high-sensitive assays permits detection of very low levels of cardiac TnT and a clinically relevant increase in TnT is stated as one that exceeds the 99th-percentile of a normal reference population [3]. HD patients, however, often have chronically elevated TnT as documented by multiple studies [4–8]. The underlying pathophysiology may reflect coronary artery disease [9, 10], subclinical myocardial injury [10], myocardial stunning [11], left ventricular (LV) hypertrophy [12], reduced renal clearance [13] and circulatory congestion [10]. The diagnosis of MI in these patients often relies on a higher cut-off value or assessment of the dynamic change in TnT, typically stated as a > 20% increase 6–9 h after presentation or by comparison with previous values [14]. In otherwise stable and asymptomatic HD patients, interpretation of elevated TnT remains a challenge for the clinician. Especially given the fact that even without suspected acute coronary syndrome higher TnT-levels are associated with a worse prognosis with a 2- to 4-fold increased 3-year mortality rate [15]. Yet, although, higher levels in cross-sectional studies are associated with worse outcome, relatively little is known about short- and long-term changes in individual HD patients. Thus, from a clinical viewpoint it is important to know the expected range, variation over time, and the impact of clinical and dialysis specific factors. In addition, few interventional studies exist. Angiotensin converting enzyme inhibitors (ACEIs) or angiotensin receptor blockers (ARBs) consistently induce strong regression of LV-hypertrophy and fibrosis [16–18]. Improvement of cardiac performance via LV-regression due to blockade of the renin-angiotensin-aldosterone system (RAAS) should thus potentially lower TnT-levels. The aim of the present study was therefore to investigate the effect of the ARB irbesartan on TnT-levels, short and long-term variation in TnT and associations with various clinical and dialysis related parameters in a cohort of newly started HD patients participating in the SAFIR study [19–22].

## Methods

### Study design

Primary results regarding residual renal function and intermediate CV endpoints have been published previously together with the study protocol [19–22]. Briefly, the SAFIR-study (acronym for” SAving residual renal Function in hemodialysis patients receiving IRbesartan) was designed as a randomized double-blind placebo-controlled multicenter trial. Inclusion criteria were dialysis vintage < 1 year, urinary output > 300 mL/day and left ventricular ejection fraction > 30% (LV EF) assessed by echocardiography. Patients, who experienced myocardial infarction or unstable angina pectoris within three months prior to admission, were excluded. Patients were recruited from six hospitals in Denmark and followed for one year. Inclusion began in May 2009 and the last patient’s last visit was in December 2012.

### Study medication and blood pressure

Patients were randomized to the ARB irbesartan 150 mg, or matching placebo. Initial dose was 150 mg/day with dosage increment after two weeks to 300 mg/day. As Irbesartan has a long plasma half-life of 15 h and is not removed by dialysis, timing of drug administration was not specified [23, 24]. Counting residual tablets monthly was used to check compliance. Patients receiving RAAS-blocking agents such as ACEI or ARB at inclusion stopped this treatment one week before baseline. A predialytic systolic blood pressure (BP) target of 140 mmHg was aimed for in all patients by adjusting dryweight and by use of all classes of antihypertensive drugs other than RAAS-blocking agents without any restrictions regarding timing and class of additional antihypertensive drugs. BP results have been published previously [19–22].

### Laboratory procedures

After 30 min of rest in the supine position, venous blood samples were drawn before HD in lithium-heparin-coated tubes. Samples were centrifuged, and the separated plasma (5 mL) was stored at − 80 °C. Plasma levels of TnT were measured with a previously validated automated Roche high sensitivity TnT immunoassay (Troponin T hs STAT Roche Diagnostics, Mannheim, Germany) on a Cobas e601 analyser according to the instructions of the manufacturer. The assay uses two cardiac TnT-specific mouse monoclonal antibodies in a sandwich format. The antibodies recognize epitopes located in the central part of the TnT molecule (amino acid positions 125–131 and 135–147, respectively). The assay does not exhibit significant cross-reaction with other troponins (skeletal muscle troponin T, cardiac/skeletal troponin I or human troponin C). Detection limit is 5 ng/L with a total imprecision of less than 10% at a level of 13 ng/L, and in 616 healthy volunteers, the upper 99th percentile was 13.5 ng/L [25]. Analytical within assay coefficient of variation in HD patients is approximately 1.7–6% according to previous studies [26–28]. N-terminal pro b-type natriuretic peptide (NT-proBNP) methodology has previously been described in detail [21].

### Arterial stiffness

Pulse wave velocity (PWV) was measured with the SphygmoCor system (version 7.0 and 8.2, Atcor Medical, Sydney, Australia) by sequential 10–20 s pulse wave recordings at the carotid artery and femoral artery using the intersecting tangent algorithm as previously described [19, 21].

### Intradialytic parameters

Intradialytic measurements of cardiac output (CO) was done within the first and the last 30 min of the dialysis session by injecting a bolus of 30 mL 37 °C isotonic saline into the venous blood line using a validated method (Hemodialysis Monitor HD02/HD03, Flow-QC tubing sets, and clip­on flow/dilution sensors Transonic Systems Inc., Ithaca, NY, USA) previously described [22]. The mean arterial blood pressure (MAP), total peripheral resistance (TPR), and stroke volume (SV) were derived by:

$$ MAP= Diastolic\  BP+\frac{1}{3}\bullet \left( Systolic\  BP- Diastolic\  BP\right) $$

$$ CO= SV\bullet Heart\ rate= MAP/ TPR $$

Intradialytic hypotension (IDH) was defined as symptomatic hypotension requiring administration of intravenous fluid or preterm ending of the dialysis session and was recorded at all dialysis sessions as previously described [22].

### Echocardiography

Echocardiography with quantification of cardiac chamber size, LV mass and function was performed as previously described [19, 21] in accordance with current guidelines [29].

### Statistics

Data were analyzed with Stata/IC 12.1 (StataCorp LP, College Station, TX 77845 USA). The assumption of normality was checked with QQ-plots, and analyses were performed using naturally log-transformed TnT due to skewness. Baseline data (qualitative variables) and various patient distributions were analyzed with χ^2^-test and continuous variables were analyzed with t-test or Wilcoxon signed-rank test. Students t-test and a multivariate repeated measurements model (xtmixed) with time and drug (placebo or ARB) and the interaction between them as factors, which allows for missing values and dropout were used for comparison of placebo vs. ARB as previously described [21, 30]. Variation over time was assessed with Bland-Altman plots and paired t-tests. Sets of duplicate log (TnT)-values (e.g. baseline vs. 1 week) were used to calculate average within- and between subject coefficients of variation (CV_1_ and CV_G_) using variance component estimates obtained by xtmixed and the following equations:

$$ {CV}_I=\left(\mathit{\exp}\left(\sqrt{SD_{Within}^2}\right)-1\right)\bullet 100\% $$

$$ {CV}_G=\left(\mathit{\exp}\left(\sqrt{SD_{Within}^2}+{SD}_{Between}^2\right)-1\right)\bullet 100\% $$

Bootstrapping was used to obtain a 95% confidence interval (95% CI) for both CV_I_ and CV_G_. Univariate and multivariate linear regression analysis was performed with baseline log (TnT) (3 multivariate models with five fixed parameters) or change (Δ = 12 months-baseline) in log (TnT) as outcome (2 multivariate models with 3 fixed parameters). Different predictors were added and tested in these models as the sixth/fourth variable. Admissions, CV-events, IDH-episodes and TnT-peaks were dichotomized to 0 or ≥ 1 events and used as outcome in univariate logistic regression analysis based on various baseline parameters and/or changes (Δ = 12 months-baseline) in TnT over time. Pearson’s *r* was used to describe linear relationships. Intention-to-treat analyses were performed and *P* < 0.05 was considered statistically significant. Values are presented as means with 95% CI unless otherwise stated. Additional details are given in the Supplement.

## Results

### Patient characteristics

**Fig. 1:**
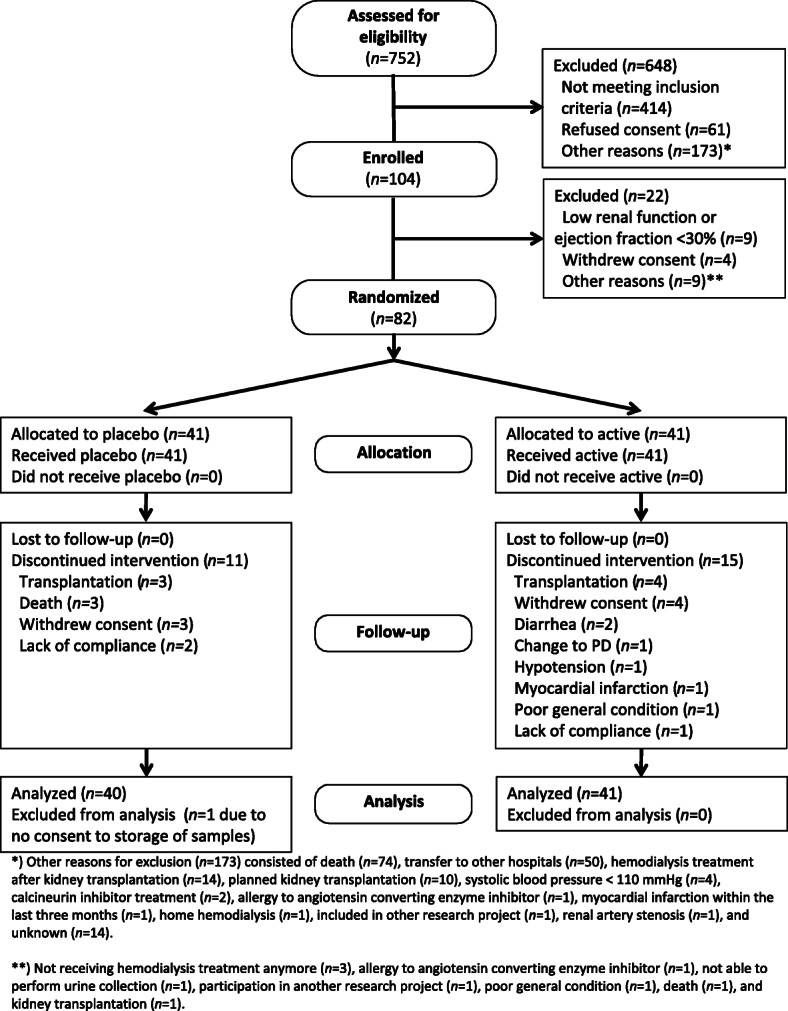
Consort Flow Chart. Inclusion and exclusion criteria have been published previously [19]. Briefly, the main inclusion criteria were urine output > 300 ml/day, dialysis vintage < 1 year and left ventricular ejection fraction > 30%

Eighty-two patients were included in the study with forty-one in each group. One patient in the placebo group did not consent to storage of plasma samples for TnT-analysis and was therefore excluded (Fig. 1). Overall, the groups were similar at baseline (Table 1). Twenty-six patients did not complete the study, eleven in the placebo and fifteen in the ARB group. Reasons for dropout were not significantly different [21].

**Table 1:** Patient characteristics

		Placebo	ARB
		**(*n*** **= 40)**	**(*n*** **= 41)**
***Demographics***			
Age	years	62 ± 14	61 ± 16
Gender (males)	n (%)	25 (63)	30 (73)
Body weight	kg	81 ± 17	79 ± 17
Body mass index	kg/m^2^	27 ± 5	26 ± 5
Diabetes	n (%)	12 (30)	13 (32)
Heart disease	n (%)	17 (43)	15 (37)
Ischemic heart disease	n (%)	9 (23)	8 (20)
Charlson co-morbidity index		3.5 ± 1.6	3.8 ± 1.9
LV mass index	g/m^2^	126 ± 36	126 ± 33
LV EF	%	60 ± 9	62 ± 9
***Cardiac medications***			
Aspirin treatment	n (%)	21 (51)	16 (39)
Antiplatelet (non-aspirin) drugs	n (%)	3 (7)	3 (7)
Statin treatment	n (%)	21 (51)	15 (37)
Nitrate medication	n (%)	6 (14)	4 (10)
***BP and BP-medication***			
Baseline systolic BP (preHD)	mmHg	145 ± 19	148 ± 21
Baseline diastolic BP (preHD)	mmHg	72 ± 12	76 ± 13
12 month systolic BP (preHD)	mmHg	136 ± 22	138 ± 20
12 month diastolic BP (preHD)	mmHg	68 ± 15	69 ± 11
BP-drugs excl. Placebo/ARB	n	2.6 ± 1.0	2.5 ± 0.9
BP-drugs excl. Placebo/ARB	DDD	1.8 ± 1.3	1.8 ± 1.2
***Dialysis parameters***			
Time on dialysis	days	141 (53–431)	148 (54–400)
AV-fistula/central catheter	n (%)	35 (88)/5 (12)	32 (78)/9 (22)
Urine output	L/24 h	1.3 ± 0.7	1.4 ± 0.8
Glomerular filtration rate	mL/min/1.73 m^2^	4.8 ± 2.3	5.7 ± 3.3
Frequency	times/week	3 (2–3)	3 (2–4)
HD-time	hours/week	10 ± 2	11 ± 3
Ultrafiltration	L	1.2 (0–4.3)	0.5 (0–3.8)
Urea reduction ratio	%	64 ± 8	62 ± 9
Hematocrit (EVF)		0.33 ± 0.05	0.34 ± 0.04

Heart disease included various conditions such as ischemic heart disease, mild heart failure (LV EF > 30%) and valvulopathy. Ischemic heart disease was defined by the presence of known ischemic heart disease, angina, pre-trial myocardial infarction, and pre-trial PCI or CABG treatment. *LV* Left ventricular; *EF* Ejection fraction; *BP* Blood pressure; *preHD* Pre-hemodialysis; *DDD* Defined daily doses; *EVF* Erythrocyte volume fraction

### Impact of ARB-treatment

**Fig. 2:**
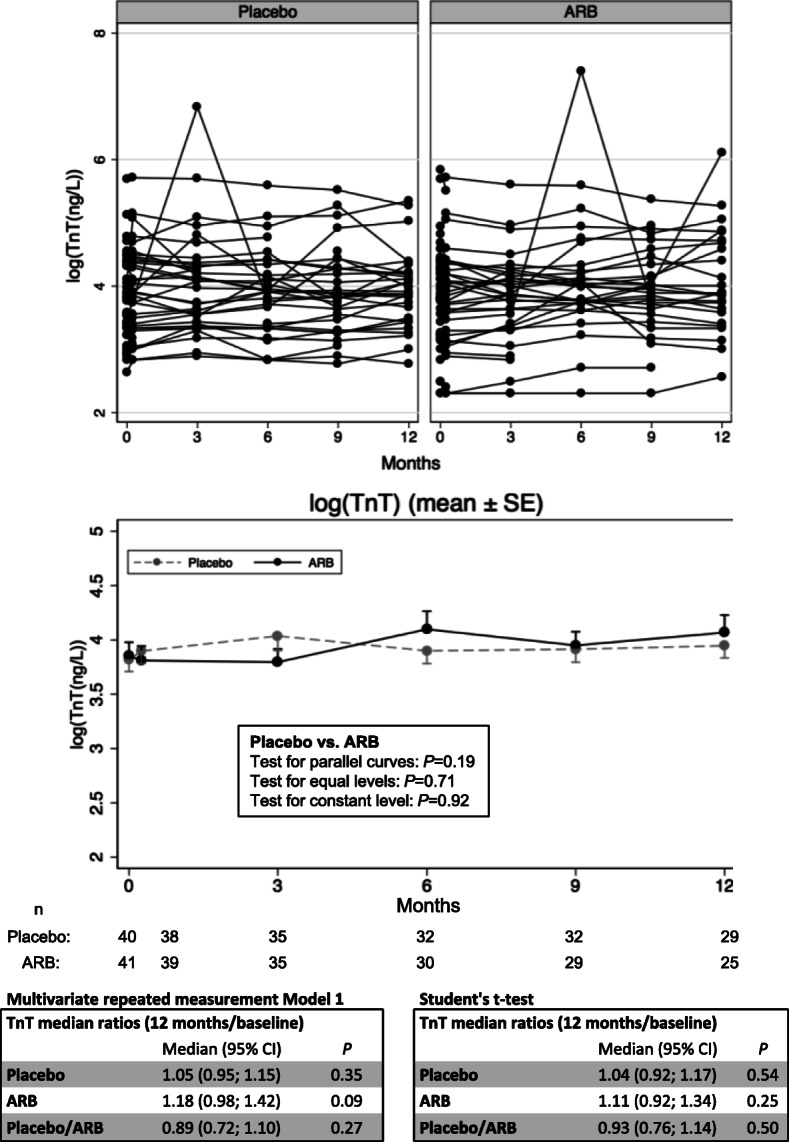
Individual and mean log-transformed TnT at various time points. Mean changes (baseline-12 months) and mean differences between groups are given as median ratios with 95% CI due to back-transformation using estimates from both xtmixed (all available patient data regardless of time in the study) and Student’s t-test thereby excluding patients with incomplete data (only patients with complete 12-months follow up). Corresponding median TnT-values and ranges (min-max) are shown in Table 2

Individual and mean changes over time in placebo and ARB-treated groups are shown in Fig. 2. Median values with ranges can be found in Table 2 in order to facilitate interpretation. There were no significant differences between the groups during the study period and ARB-treatment did not significantly affect mean TnT-levels over the 12-month study period (*P* ≥ 0.19 in all tests for parallel curves, equal levels, and constant levels) as shown in Fig. 2. Changes between baseline and 12 months are given as median ratios (12 months/baseline) with 95% confidence intervals (95% CI) due to back-transformation from natural log-transformed mean values in the placebo and ARB group. Overall, median ratios were not significantly different, neither when using all available data (estimates from the multivariate repeated measurement model 1), nor when excluding patients with incomplete data (estimates based on Student’s t-test) as shown in Fig. 2. CV-events, admissions and IDH episodes were not significantly different after 12 months and total number of events in the two groups (placebo/ARB) were: 90/61 (Admissions); 18/14 (CV-events) and 50/63 (IDH-events). CV-events consisted of (placebo/ARB): MI: 0/2; angina: 8/5; percutaneous coronary intervention (PCI): 2/1; coronary artery bypass grafting (CABG): 0/1; arrythmia: 4/3 and valvular diseases: 4/2.

**Table 2:** TnT median values with ranges

			TnT			TnT
Time	Group	n	ng/L	Group	n	ng/L
**Baseline**	Placebo	40	45 (14–295)	All	81	45 (10–343)
	ARB	41	46 (10–343)			
**1 week**	Placebo	38	48 (17–303)	All	77	47 (10–305
	ARB	39	47 (10–305)			
**3 months**	Placebo	35	59 (18–927)	All	70	51 (10–927)
	ARB	35	48 (10–271)			
**6 months**	Placebo	32	50 (17–267)	All	62	52 (10–1642)
	ARB	30	56 (10–1642)			
**9 months**	Placebo	32	46 (16–249)	All	61	48 (10–249)
	ARB	29	52 (10–214)			
**12 months**	Placebo	29	50 (16–210)	All	54	49 (13–451)
	ARB	25	49 (13–451)			

*TnT* Troponin T. Values are presented as median with range (min-max). Statistical analysis was performed after logarithmic transformation due to skewed data

### Variation in TnT over time

**Fig. 3:**
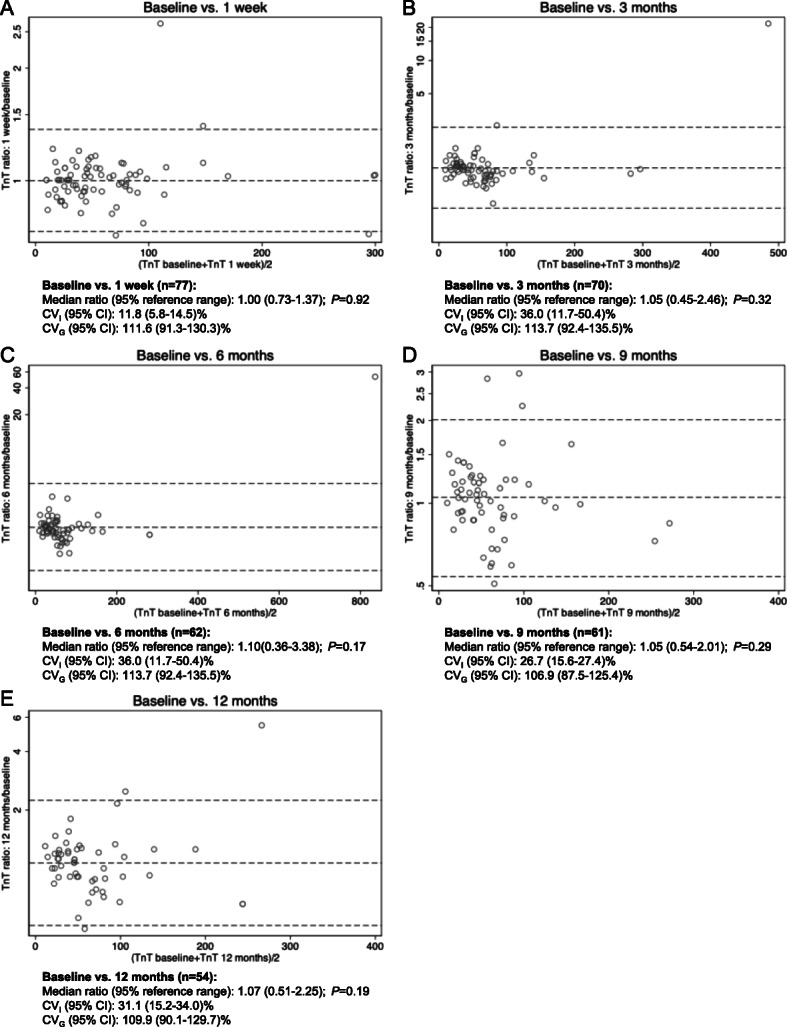
Bland-Altman plots (**a**-**e**) showing variation in TnT over time (baseline vs. subsequent measurements). Median ratios due to back-transformation with 95% limits of agreement (reference range for difference) and corresponding within-subject (CV_I_) & between-subject (CV_G_) coefficients of variation with 95% confidence intervals (95% CI) are also given. Note logarithmic scale on Y-axis in all Bland-Altman plots

**Fig. 4:**
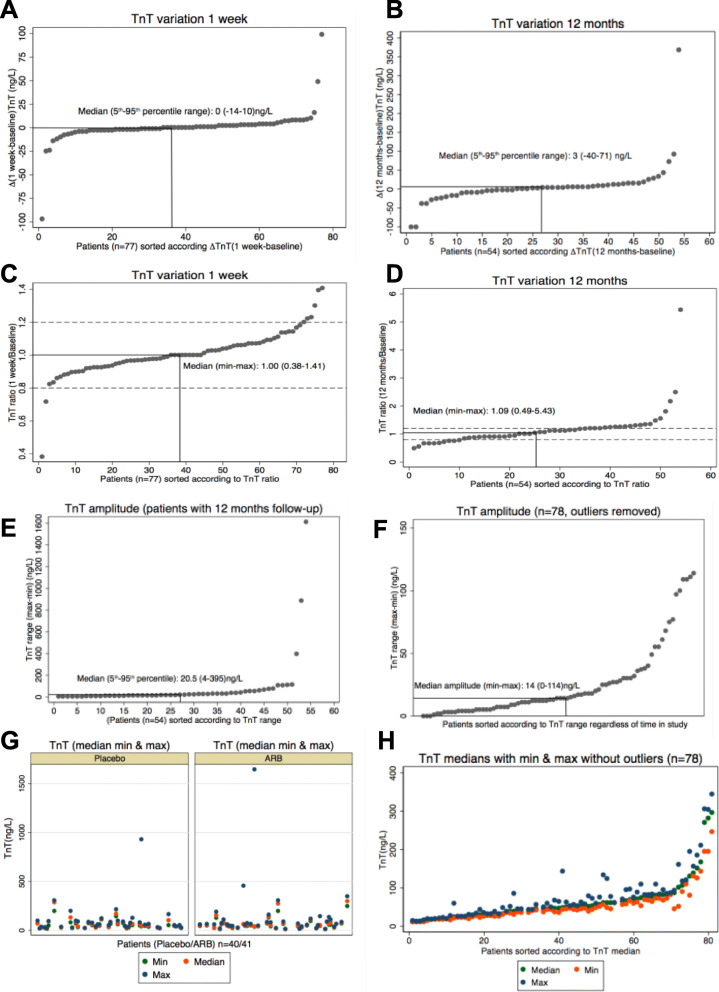
**a** Shows short-term TnT variation baseline vs. 1 week with patients sorted according to magnitude of change in TnT (lowest to highest). **b** Long-term TnT variation baseline vs. 12 months with individual patients sorted according to magnitude of change (lowest to highest). **c** Similar to A but with patients sorted according to magnitude of TnT ratio (1 week/baseline). **d** Similar to B but with patients sorted according to magnitude of TnT ratio (12 months/baseline). **e** TnT amplitude (max-min) from patients with complete 12 months follow-up with patients sorted according to magnitude of change (lowest to highest). **f** TnT amplitude (max-min) from all patients, except outliers, regardless of time in the study with patients sorted according to magnitude of change (lowest to highest). **g** Range (min & max) and median TnT from all patients sorted according to intervention (Placebo/ARB) regardless of time in the study. **h** Range (min & max) and median TnT from all patients, except outliers, sorted according to median TnT regardless of time in the study

Since ARB-treatment had no significant impact on TnT-levels, all samples were pooled into one group regardless of treatment status for analysis of variation over time. Figure 3 shows Bland-Altman plots, median ratios and corresponding within-subject (CV_I_) and between-subject (CV_G_) coefficients of variation regardless of treatment status. Median ratios with 95% limits of agreement (reference range for difference) were: 1.00(0.73–1.37); *P* = 0.92 (Baseline vs. 1 week) and 1.07(0.52–2.25); *P* = 0.19 (Baseline vs. 12 months), respectively. Corresponding CV_I_ and CV_G_ (95% CI) were: CV_I_: 11.8 (5.8–14.5)%; CV_G_ 111.6 (91.3–130.3)% (Baseline vs. 1 week) and CV_I_: 31.1 (15.2–34.0)%; CV_G_: 109.9 (90.1–129.7)% (Baseline vs. 12 months). Median change (min; max) after 12 months was: 3(− 101; 368) ng/L (all samples; *n* = 54). Median (5th–95th-percentile range) week-to-week and one-year individual TnT-variation were: 0(− 14–10) ng/L (baseline-1 week: *n* = 77) and 3(− 40–71) ng/L (baseline-12 months: *n* = 54) as shown in Fig. 4a-b. Using a 20% change in TnT as cut-off (typically used for MI-diagnosis), 7.8% (6/77 patients) had > 20% increase in TnT and 2.6% (2/77 patients) had > 20% decrease in TnT one week after baseline. At 12 months 20.4% (11/54 patients) had > 20% increase in TnT and 22.2% (12/54 patients had > 20% decrease in TnT (Fig. 4c-d). Median (5-95th-percentile range) TnT amplitude in patients with 12-months of follow-up (*n* = 54) was 20.5(4–395) ng/L (Fig. 4e). Using data from all patients regardless of time in the study (*n* = 81), individual median amplitude (max-min) over the entire 12-month period was 14(0–1611) ng/L corresponding to range/median 30(0–4131)%. After removal of 3 outliers (TnT > 400 ng/L) individual median amplitude (min-max) over the entire 12-month period was 14(0–114) ng/L (Fig. 4f) corresponding to range/median 29(0–208)%. Using only samples from patients with complete 12-months follow-up (n = 54), individual median amplitude (max-min) over the entire 12-month period was 20.5(3–1611) ng/L corresponding to range/median 38(15–4131)%. After removal of 3 outliers (TnT > 400 ng/L) individual median amplitude (min-max) over the entire 12-month period was 18(3–114) ng/L corresponding to range/median 36(15–165)%. Most patients exhibited minor TnT-fluctuations close to the median. However, a low median TnT-level did not exclude subsequent rise in TnT (Fig. 4g-h).

### Baseline TnT correlations (univariate analysis)

**Fig. 5:**
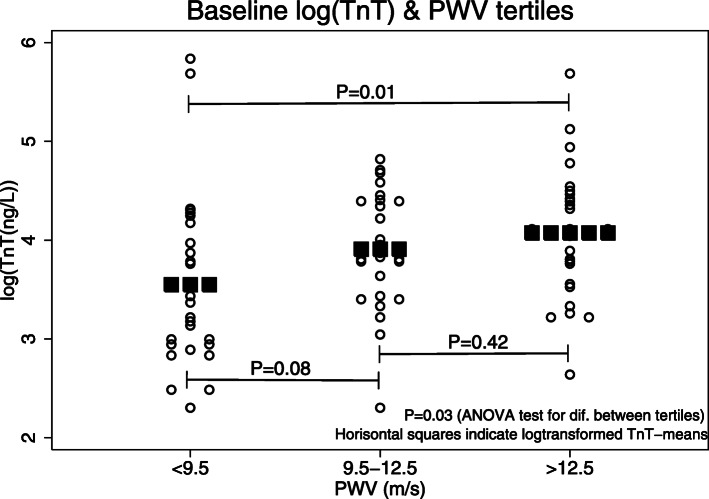
Baseline pulse wave velocity (PWV) tertiles and log-transformed baseline TnT. The geometric baseline TnT-means with 95% confidence intervals (95%-CI) were: cfPWV< 9.5 m/s (*n* = 27): 35(26–56) ng/L; cfPWV 9.5–12.5 m/s (*n* = 26): 50(38–66) ng/L; cfPWV> 12.5 m/s (n = 26): 59(44–78) ng/L.

Baseline log-transformed TnT was positively correlated with age, diabetes, Charlson comorbidity index, Ultrafiltration (UF) volume, arterial stiffness (PWV and PWV-tertiles)), change in intradialytic total peripheral resistance (ΔTPR = TPR_end_-TPR_start_) and NT-proBNP. Baseline TnT was negatively correlated with change in intradialytic cardiac output (ΔCO=CO_end_-CO_start_), hematocrit and residual renal function (urine volume and GFR) as shown in Table 3. Echocardiographic parameters such as LV mass index and LV EF were not significantly associated with baseline TnT in univariate analysis. The impact of arterial stiffness on baseline TnT was examined further by splitting baseline PWV into tertiles as shown in Fig. 5. Known heart disease at baseline was only borderline significant (*P* = 0.06). Multivariate regression analysis was also performed with baseline log (TnT) as outcome and results are shown in the Supplement (Table S1).

**Table 3:** Univariate regression analysis based on baseline log (TnT)

Parameter		n	β (95% CI)	*P*	r^2^
Age (years)		81	0.01 (0.00; 0.02)	**< 0.05**	0.05
Female gender		81	−0.25(−0.60; 0.10)	0.15	0.03
Cornell (S_V3_ + R_aVL_) (mm)		81	0.02 (0.00; 0.03)	0.08	0.04
Diabetes		81	0.45 (0.11; 0.80)	**0.01**	0.08
Heart disease		81	0.32(−0.01; 0.65)	0.06	0.04
Charlson comorbidity index		81	0.13 (0.05; 0.22)	**0.004**	0.10
GFR (mL/min/1.73m^2^)		77	−0.07(− 0.13; − 0.01)	**0.02**	0.07
Urine output (L/24 h)		79	−0.35(− 0.57; − 0.14)	**0.001**	0.12
Ultrafiltration (L)		81	0.23 (0.11; 0.35)	**< 0.001**	0.16
PWV (m/s)		79	0.05 (0.00; 0.10)	**0.04**	0.05
PWV-tertiles (m/s)	9.5–12.5	26	0.36(−0.04; 0.76)	0.08	0.09
(PWV < 9.5 m/s = ref.)	> 12.5	26	0.52 (0.13; 0.92)	**0.01**	
LV mass index (g/m^2^)		80	0.00 (0.00; 0.01)	0.08	0.04
LV EF (%)		80	−0.01(−0.03; 0.01)	0.20	0.02
ΔCO (L/min)		62	−0.23(− 0.39; − 0.07)	**0.007**	0.12
ΔTPR (mmHg/(L/min))		61	0.07 (0.01; 0.12)	**0.03**	0.08
log (NT-proBNP) (log (nmol/L))		81	0.27 (0.14; 0.39)	**< 0.001**	0.19
Haematocrit (EVF)		81	−5.10(−8.68; −1.52)	**0.006**	0.09

*TnT* Troponin T; *GFR* Glomerular filtration rate; *PWV* Carotid-femoral pulse wave velocity; *LV* Left ventricular; *EF* Ejection fraction; *ΔCO* Change in intradialytic cardiac output (ΔCO = CO_end_-CO_start_); *ΔTPR* Change in intradialytic total peripheral resistance ((ΔTPR = TPR_end_-TPR_start_); *NT-proBNP* N-terminal pro b-type natriuretic peptide; *EVF* Erythrocyte volume fraction

### Prediction of clinical events based on baseline TnT

High baseline TnT increased the risk of admission and CV-events (0 vs. ≥1 admissions/CV-events) during follow-up with natural log-transformed TnT odds-ratios (ORs): 2.62(1.22–5.64); *P* = 0.01 and 2.25(1.04–4.86); *P* = 0.04, respectively (Table 5). Baseline TnT was also borderline significant in terms of predicting IDH-events (0 vs. ≥1 events) during follow-up with natural log-transformed TnT OR: 1.87(0.98–3.59); *P* = 0.06.

### Parameters associated with change in TnT

Increase in TnT over time (Δ = 12-months-baseline) was significantly associated with increase in LV mass and NT-proBNP and decrease in LV EF and late intradialytic stroke volume 30 min before end of HD (ΔSV2) in univariate analysis (Table 4). Results from multivariate analysis are shown in the Supplement (Table S2).

**Table 4:** Univariate regression analysis with change in log (TnT) (Δ = 12 months-baseline)

Parameter		n	β (95% CI)	*P*	r^2^
Age at baseline		54	0.005(−0.002; 0.013)	0.13	0.04
Heart disease		54	0.22 (0.02; 0.42)	**0.03**	0.08
Diabetes		54	−0.12(−0.34; 0.09)	0.25	0.03
Charlson comorbidity index		54	−0.01(− 0.07; 0.06)	0.87	0.00
ΔGFR (mL/min/1.73m^2^)		49	−0.03(− 0.10; 0.04)	0.40	0.01
ΔUrine output (L/24 h)		52	0.04(−0.13; 0.21)	0.63	0.00
ΔUltrafiltration (L)		53	−0.02(− 0.12; 0.08)	0.68	0.00
Baseline Cornell (S_V3_ + R_aVL_) (mm)		54	0.012 (0.001; 0.022)	**0.03**	0.09
ΔCornell (S_V3_ + R_aVL_) (mm)		54	−0.01(−0.02; 0.01)	0.38	0.02
Baseline PWV (m/s)		53	0.01(−0.02; 0.04)	0.55	0.01
Baseline PWV-tertiles (m/s)	9.5–12.5	27	0.09(−0.19; 0.37)	0.52	0.02
(PWV < 9.5 m/s = ref.)	> 12.5	26	0.13(−0.13; 0.38)	0.32	
ΔPWV (m/s)		52	0.01(−0.05; 0.06)	0.74	0.00
ΔLV mass index (g/m^2^)		53	0.003 (0.001; 0.006)	**0.01**	0.11
ΔLV EF(%)		53	−0.01(−0.02; 0.00)	**0.01**	0.12
ΔSV2 (mL)		41	−0.01(− 0.01; 0.00)	**0.04**	0.11
Δlog (NT-proBNP) (log (nmol/L))		54	0.18 (0.09; 0.27)	**< 0.001**	0.25
ΔHematocrit (EVF)		54	−0.93(−2.94; 1.09)	0.36	0.02

Abbreviations: *TnT* Troponin T; *GFR* Glomerular filtration rate; *PWV* Carotid-femoral pulse wave velocity; *LV* Left ventricular; EF: Ejection fraction; *SV2* Late intradialytic stroke volume 30 min before end of HD; *NT-proBNP* N-terminal pro b-type natriuretic peptide; *EVF* Erythrocyte volume fraction

### Changes in TnT over time and correlations with clinical outcome

Three different approaches were used to investigate the relationship between change in TnT over time and clinical outcome in terms of hospital admissions, CV-events and IDH episodes (Table 5).

**Table 5:** Univariate logistic regression results

		CV-events (0 vs. ≥1)	Admissions (0 vs. ≥1)	IDH-episodes (0 vs. ≥1)	TnT-peaks (0 vs. ≥1)
	n	OR (95% CI)	OR (95% CI)	OR (95% CI)	OR (95% CI)
***Baseline TnT***					
log (TnT)	81	**2.25 (1.04–4.86)**	**2.62 (1.22–5.64)**	1.87 (0.98–3.59)	0.94 (0.52–1.71)
Low (TnT ≤ 45 ng/L) vs. High (TnT > 45 ng/L)	81	1.72 (0.58–5.09)	**3.74 (1.29–10.86)**	**2.68 (1.05–6.79)**	0.84 (0.35–2.03)
***TnT change (Δ = 12 months-baseline)***					
Δlog (TnT)	54	1.17 (0.20–6.93)	0.64 (0.14–2.91)	0.26 (0.04–1.56)	–
Increase (ΔTnT> 0) vs. Decrease (ΔTnT≤0)	54	0.76 (0.19–3.01)	0.30 (0.08–1.10)	**0.31 (0.10–0.96)**	–
***TnT amplitude (max-min)***					
Low (TnT ≤ 20.5 ng/L) vs. High (TnT > 20.5 ng/L)	54	0.61 (0.15–2.46)	**4.60 (1.24–16.97)**	3.08 (0.97–9.67)	–
***TnT-Peaks***					
No peak vs. ≥1 peak	81	1.05 (0.51–2.15)	2.30 (1.00–5.30)	1.33 (0.73–2.44)	–
***Asprin (n = 37)*** **vs.** ***non-aspirin treatment (n = 44)***	81	1.96 (0.66–5.80)	0.54 (0.20–1.44)	0.69 (0.28–1.73)	**0.28 (0.11–0.72)**

Abbreviations: *TnT* Troponin T; *TnT-peak* 20% increase above individual TnT median value; *CV* Cardiovascular; *IDH* Intradialytic hypotensive episodes defined as symptomatic hypotension requiring administration of intravenous fluid or preterm ending of the dialysis session

Regardless of whether change in TnT (Δ = 12 months-baseline) was assessed as Δlog (TnT) or as a dichotomized outcome increase (ΔTnT> 0) vs. decrease (ΔTnT≤0) it was not significantly associated with admissions and CV-events during follow-up. In logistic regression analysis, TnT-increase after 12-months was associated with a lower risk of IDH-events with (TnT-increase vs. TnT-decrease) OR: 0.31(0.10–0.96); *P* = 0.04.

### TnT-amplitude

TnT-amplitude (max-min) was used to capture the change from the lowest to the highest TnT-value during the entire study period. If analysis was restricted to patients with complete 12-months follow-up (*n* = 54), the median TnT-amplitude was 38% or 20.5 ng/L as previously mentioned. By dichotomizing the amplitude into low (TnT ≤ 20.5 ng/L) or high (TnT > 20.5 ng/L), high TnT-amplitude was significantly associated with increased number of admissions, borderline significant in terms of IDH episodes and non-significant in terms of CV-events (Table 5). Using univariate logistic regression analysis, ORs (0 vs. ≥ 1 event) for comparison of high vs. low TnT-amplitude were: 4.60(1.24–16.97); *P* = 0.02 (admissions) and 3.08(0.97; 9.67); *P* = 0.05 (IDH-episodes).

### TnT-peak frequency

The number of TnT-peaks (defined as 20% increase above the individual patient TnT-median calculated from all available samples regardless of time in the study) were assessed. TnT-peak distribution in our cohort was (number of patients): No peak: 46(57%); 1 peak: 25(31%); 2 peaks: 9(11%); and 3 peaks: 1(1%). Baseline TnT-level, ARB-treatment, known heart disease, diabetes and arterial stiffness (baseline PWV-tertiles) had no significant impact on peak frequency. In univariate logistic regression analysis, the risk of admission tended to increase with the number of TnT-peaks with OR (0 vs. ≥ 1 admission) 2.30(1.00–5.30); *P* = 0.05. The frequency of CV-events or IDH-episodes was not significantly different when comparing patients without peaks to those with peaks (Table 5).

### Aspirin vs. non-aspirin treatment

In univariate logistic regression analysis, aspirin treatment decreased the risk of TnT-peaks (0 vs. ≥ 1 TnT peak) with OR: 0.28(0.11–0.72); *P* = 0.008. Admissions, CV and IDH events were not significantly different in aspirin vs. non aspirin treated. Additional details are given in the Supplement.

## Discussion

This study found no significant impact of long-term treatment with the ARB irbesartan on predialytic TnT-levels in HD patients. Overall, TnT was quite stable with an individual median ratio over the entire 12-month period with 95% limits of agreement of 1.07(0.51–2.25). Yet, during 12 months of observation some patients exhibited a significant rise in TnT and a low median TnT-level did not exclude subsequent rise in TnT. Our study investigated various clinical and dialysis related parameters associated with TnT. Diabetes, UF volume, arterial stiffness (PWV), change in intradialytic total peripheral resistance and NT-proBNP were positively correlated with baseline TnT whereas hematocrit, residual renal function (GFR or urine volume) and change in intradialytic cardiac output were negatively correlated with baseline TnT. Patients with preserved renal function are less prone to volume overload possibly explaining why TnT was lower in these patients in line with the positive correlations found between TnT and UF volume and NT-proBNP, respectively. Moreover, a better clearance of TnT fragments in patients with preserved renal function could also be a relevant factor to consider, as demonstrated by a previous study [13].

The strengths of this study include serial measurements which allowed us to describe both short (one week) and long-term (12 months) TnT-changes in our cohort. Within-subject and between-subject coefficients of variation were similar to previous studies in HD patients using high-sensitivity TnT-assays when comparing short-term estimates [27, 28]. Unlike most previous studies our study included intervention with an ARB, and patients were well characterized regarding cardiac status (e.g. LV mass, LV EF and NT-proBNP), arterial stiffness, intradialytic hemodynamics, medications and clinical events. To the best of our knowledge, our study is the first to examine the impact of long-term ARB-treatment on TnT-levels in HD patients in a randomized double-blind placebo-controlled design.

LV hypertrophy (LVH) is frequent in end-stage renal disease (ESRD) [31–33] and with manifest LVH, myocardial capillary growth is expected to lack behind cardiomyocyte hypertrophy causing cardiomyocyte/capillary mismatch leading to increased oxygen diffusion distance, reduced ischemic tolerance of the heart, which in turns leads to subclinical ischemia of the myocardium and thereby amplified leakage of cardiac troponins including TnT [10]. RAAS-blocking agents such as ACEI or ARB are generally considered to be beneficial in terms of regression of LVH [18] and improvement in LV EF [34]. As previously reported [21], we found no significant effect of ARB-treatment on BP, LV mass and LV EF and our patients did not exhibit pronounced LVH or heart failure, which may explain why there was no significant impact of ARB-treatment on TnT in our study. Not many studies have examined long-term changes in TnT with serial measurements beyond six months like our study. Conway et al. examined 75 HD patients out of which 46 completed 4 serial pre- and post HD TnT measurements after 15 months [35]. TnT was frequently elevated and baseline TnT-levels were associated with an increased risk of mortality and acute coronary syndrome. Bloch et al. followed 238 HD patients out of which 164 completed 24 months of follow-up using pre-HD TnT-measurements at baseline, 18 and 24 months, respectively [4]. TnT increased by 50% in < 1/3 of patients and doubled in only 10% of patients during 18- and 22-months follow-up. Baseline TnT was a significant predictor of all cause and CV death. Mongeon et al. followed 100 HD patients out of which 78 completed 12 months of follow-up using both pre- and post-HD measurements at baseline, 6 months and 12 months, respectively [36]. TnT was found to be stable over a 12-month period although levels tended to increase more between 6 and 12 months. Pre- and post-HD levels were similar, but higher TnT-levels were found in patients with coronary artery disease. Finally, Roberts et al. studied the impact of carvedilol vs. placebo on TnT in a mixed cohort of 72 patients including both HD and peritoneal dialysis (PD) patients [37]. TnT was measured at baseline, 6 months and 12 months, respectively. Forty-nine patients completed run-in and 31 completed 12-month follow-up. TnT-levels at baseline and during follow-up were similar to our study and there was no significant change in mean TnT-levels +/− Carvedilol treatment. Individual variation and correlation with clinical outcome were not reported. Our study thus adds significantly to our understanding of especially the temporal variation and on the impact of ARB-treatment on TNT in HD-patients. High baseline TnT was in our study associated with a higher risk of admission and CV-events during follow-up. Elevated TnT is a well-known risk factor for negative outcome even when MI is not suspected in large cohorts of HD patients [15]. Our findings are in line with this although strictly speaking our study was not powered for hard endpoints. Moreover, our study was able to demonstrate that rise in TnT over 12 months was significantly correlated with deterioration of cardiac status (increase in LV mass and NT-proBNP and decrease in LV ejection fraction and late intradialytic stroke volume). Despite this, rise in TnT after 12 months was not significantly associated with admissions, CV or IDH events during follow-up. We suspect this could be due to the relatively low number of patients in our study. We did demonstrate that a high TnT-amplitude (> 20.5 ng/L) was significantly associated with increased number of admissions and borderline significant in terms of IDH-episodes suggesting a link between IDH and myocardial damage in accordance with a previous study [38]. Similarly, we found that admissions tended to be more likely in patients with ≥1 peak in TnT (defined as a 20% increase above the individual patient TnT-median) compared to non-peakers.

The relatively low TnT-levels in our study suggest occurrence of predominately minor myocyte injury. Previous studies investigating the impact of dialysis on TnT-levels showed that TnT may increase after HD due to hemoconcentration [35] but generally reported little change [5, 36] or even a slight reduction in TnT after HD [39]. Nevertheless, the hemodynamic stress associated with dialysis including fluctuations in electrolytes and large UF volumes could be associated with a transient increase in TnT and repeated HD sessions could lead to a progressive increase in TnT over time in some patients as previously reported [4, 35, 36].

The relationship between increased arterial stiffness, a hallmark of ESRD, and TnT-levels was a novel finding which may reflect compromised myocardial perfusion due to early return of the arterial pulse wave during systole rather than diastole [40]. Increased arterial stiffness and low hematocrit combined with LVH and a high prevalence of coronary artery disease substantially augments the risk of ischemia and TnT-release. Impairment of myocardial function induced by dialysis treatment, known as cardiac stunning, may also contribute [11]. Jefferies et al. demonstrated that the prevalence of myocardial stunning can be reduced with increasing intensity (frequency and duration) of HD and that there was a strong positive relationship between UF rate and severity of stunning as well as a tendency towards lower levels of TnT with frequent dialysis [41]. Our study found that TnT-levels tended to increase with increased UF volume and a drop in intradialytic cardiac output. Since our study did not include home dialysis patients, we could not explore trends regarding HD frequency.

Interestingly, we found that use of aspirin vs. non-use was associated with fewer TnT-peaks during follow-up. There is a paucity of definitive data concerning the efficacy of aspirin in dialysis patients and most observational studies suffer from confounding by indication explaining why some studies found aspirin use to be associated with increased CV mortality or adverse CV-events [42–44]. Our study, although not designed to study the effects of aspirin, suggest that aspirin may be beneficial in terms of preventing asymptomatic ischemia in HD patients consistent with the Kidney Disease Outcomes Quality Initiative (KDOQI) clinical practice guideline [45].

### Clinical implications

Despite the fact the TnT is a strong marker for poor outcome, the usefulness of TnT sampling in dialysis patients could be questioned. So far, there are no intervention studies that compare treatment strategies stratified by TnT levels in dialysis patients without MI. Our study showed that a rise in TnT reflected deterioration of cardiac function, indicating that frequent TnT monitoring could be clinically relevant. From a pragmatic point of view, measuring TnT e.g. twice yearly gives each patient’s baseline TnT-values for comparison when acute MI is suspected and may improve the clinician’s ability to diagnose deterioration of cardiac function in otherwise asymptomatic patients and facilitate pre-emptive cardiac evaluation with additional screening (stress testing, echocardiography, coronary angiography) or intensified prevention strategies (platelet inhibitors, anemia correction, correction of volume overload). This approach could be tested in future studies and may potentially alleviate CV disease burden in dialysis patients alongside clarifying the added value of frequent TnT sampling.

### Limitations

First of all, our findings are limited to HD patients without recent episodes of angina or MI, no heart failure, with some preserved renal function and a relatively short time on dialysis. CV disease and instability during HD may be more prevalent among more morbid and fragile patients. We did not collect blood samples after dialysis and our results therefore reflect the predialytic state. In addition, samples were not collected based on suspicion of myocardial ischemia and may therefore underestimate the true variance. Due to preserved urine output in the majority of our patients, relatively small UF volumes were prescribed during HD compared to other studies [46, 47]. In HD patients with more pronounced CV disease or larger fluid fluctuations, the fluctuations in TnT may differ as well as the response to ARB-treatment. In our cohort, ARB-treatment did not significantly reduce BP and results could have been different in the presence of a BP difference. Patients treated with PD may respond differently in terms of TnT-fluctuations and response to ARB [48].

## Conclusions

The ARB irbesartan had no significant impact on predialytic TnT-levels. Week-to-week TnT-variation was low, yet over 12 months individual patients had considerable TnT fluctuations. The median TnT-amplitude, capturing the change from the lowest to the highest TnT-value observed during the 12 months study period was 38% or 20.5 ng/L. High TnT at baseline was associated with a higher risk of admission and CV-events during follow-up. Rise in TnT over time was significantly correlated with markers of cardiac deterioration and admissions during follow-up were significantly more likely with a high (TnT-amplitude> 20.5 ng/L) than a low TnT-amplitude. Aspirin use was associated with fewer peaks in TnT and may prove beneficial in terms of preventing cardiac damage in HD patients. Regular monitoring of TnT may improve the ability to diagnose deterioration of cardiac status in otherwise asymptomatic HD patients but requires further studies prior to implementation into clinical praxis.

## Supplementary information

**Additional file 1.** Methodology. Additional results, Table S1, Table S2

## Data Availability

The datasets that support the findings of the current study are available from the corresponding author on reasonable request.

## Abbreviations

ACEI: Angiotensin converting enzyme inhibitor
ARB: Angiotensin II receptor blocker
BP: Blood pressure
CABG: Coronary artery bypass grafting
CO: Cardiac output
ΔCO: Change in intradialytic cardiac output (ΔCO = CO_end_-CO_start_)
CV: Cardiovascular
CV_G_: Between-subject coefficient of variation
CV_I_: Within-subject coefficient of variation
DDD: Defined daily doses
EF: Ejection fraction
EVF: Erythrocyte volume fraction
ESRD: End-stage renal disease
GFR: Glomerular filtration rate
HD: Hemodialysis
IDH: Intradialytic hypotensive episodes
LV: Left ventricular
LVH: LV hypertrophy
MAP: Mean arterial blood pressure
MI: Myocardial infarction
NT-proBNP: N-terminal pro b-type natriuretic peptide
OR: Odds ratio
PCI: Percutaneous coronary intervention
PD: Peritoneal dialysis
preHD: Pre-hemodialysis
PWV: Carotid-femoral pulse wave velocity
RAAS: Renin-angiotensin-aldosterone system
SAFIR: Acronym for SAving residual renal Function in hemodialysis patients receiving Irbesartan
SV: Stroke volume
SV1: Early intradialytic stroke volume within 30 min after start of HD
SV2: Late intradialytic stroke volume 30 min before end of HD
TnT: Troponin T
TPR: Total peripheral resistance
ΔTPR: Change in intradialytic total peripheral resistance ((ΔTPR = TPR_end_-TPR_start_)
UF: Ultrafiltration
