# Supplementary material for: High-sensitivity Troponin T in hemodialysis patients: a randomized placebo-controlled sub-study investigating angiotensin-II-blockade, variation over time and associations with clinical outcome
Source: BMC Nephrol. 2020 Oct 28;21:452. doi: 10.1186/s12882-020-02103-1 (PMC7594269; doi:10.1186/s12882-020-02103-1)
Supplement: Supplementary file 1 — Additional file 1. Methodology. Additional results, Table S1, Table S2 [file 12882_2020_2103_MOESM1_ESM.docx]

**Supplementary Material**

Methodology
Additional results
Table S1
Table S2

**Methodology**

*Statistical details*Log-transformed TnT-data were analyzed based on a multivariate repeated measurements model (xtmixed) with visit (baseline, 1 week, 3 months, 6 months, 9 months, and 12 months) and drug (placebo or ARB) and the interaction between them as factors which allows for missing values and dropout in the sense that it only excludes patients if all observations are missing for that patient. The mixed effects model is a population model in its nature. Observations from patients who complete the study carry the most weight in the estimation of what happens in the full twelve-month period. Those, who do not complete the study, contribute with valuable information until they drop out. Statistical analysis was performed after logarithmic transformation due to skewed data. An approximate test for the hypothesis of equal standard deviations and correlations in the two groups was performed and the analysis was adjusted according to whether or not equal standard deviations and correlations were achieved. Model validation was performed by comparing observed and modeled within-subject standard deviations and correlations and by inspecting QQ-plots. Consequently, the two groups were compared regarding the development over time using four different models:
Model 1: Different development over time
Model 2: Parallel curves (same development over time)
Model 3: Equal levels in the two groups
Model 4: Constant curves (no change over time).
A likelihood ratio test (LR-test) was used to compare the models in order to describe the development over time. The first test compared Model 1 with Model 2. If the test was non-significant, we assumed parallel curves (same development over time). In case of parallel curves, we proceeded testing whether equal levels could be assumed. This was done by testing Model 2 versus Model 3. Finally, in case of equal levels in the two groups we tested whether there was a change over time (constant curves) by comparing Model 3 with Model 4. Pairwise comparisons between and within the placebo and ARB group were based on estimates from Model 1. Mean changes (baseline-12 months) and mean differences between groups were analyzed by using estimates from Model 1 and also by Student’s t-test thereby excluding patients with incomplete data.

**Additional results***Blood pressure and impact of ARB-treatment*

Predialytic BP decreased to similar levels during the 12-month study period Δ(12-month-baseline)preHD systolic mean BP (Placebo/ARB): -8/-10 mmHg; *P*=0.8), use of additional antihypertensive medication besides placebo/ARB, ultrafiltration (UF) volume, and dialysis dosage were not significantly different and adverse events were also similar in the two groups as previously reported (1, 2).

*Baseline TnT correlations (multivariate analysis)*

Multivariate regression analysis was also performed with baseline logtransformed TnT as outcome (Table S1). UF volume was closely inversely related to residual renal function (GFR and urine output) and Charlson comorbidity index was closely linked with comorbidities such as heart disease and diabetes. Three different multivariate models were therefore constructed and used for multivariate regression analysis. Each model consisted of five fixed variables as indicated and various predictors were added and tested as the sixth variable. Haematocrit (EVF), log(NT-proBNP) and arterial stiffness (PWV-tertiles) remained significantly correlated with baseline TnT in multivariate analysis regardless of the model used. Age and intradialytic parameters (ΔCO and ΔTPR) were not significant in multivariate analysis. LV EF was significantly negatively correlated with baseline TnT when added as the sixth parameter to Model 1 and Model 3. Diabetes and known heart disease were also significant in multivariate regression analysis (Model 2 and Model 3, respectively). LV mass was not significantly associated with baseline TnT and Cornell voltage as an ECG-based measure of LV hypertrophy was only significant in Model 3.

*Baseline TnT and IDH-episodes*
If baseline TnT was dichotomised based on the baseline median TnT-value of 45 ng/L into low (≤45 ng/L) and high (>45 ng/L) significantly more patients in the high-level group experienced IDH-events during follow-up (patients with ≥1 IDH-events): 19/39 (49%) in the TnT>45 ng/L group vs. 11/42 (26%) in the TnT≤45 ng/L group; *P*=0.04 (χ2-test). In univariate logistic regression analysis TnT>45 ng/L at baseline was associated with a higher risk of IDH-events during follow-up with OR: 2.68(1.05-6.79); *P*=0.04. However, in multivariate analysis with additional adjustment for LV mass, UF, p-albumin, antihypertensive drugs and time in the study it was no longer significant and adjusted OR (high vs. low TnT) was: 2.37(0.77-7.33); *P*=0.14.

*Twelve-month change in TnT (multivariate analysis)*Change in NT-proBNP was significantly positively correlated with change in LV mass (*r*^2^=0.25; *P*<0.001) and two models with either Δlog(NT-proBNP) (Model 1) or ΔLV mass index (Model 2) was therefore used with Δlog(TnT) as outcome. Both models used ΔSV2 and known heart disease as fixed variables and various predictors were added as the fourth variable (Table S2). In model 1, Δlog(NT-proBNP) remained significant and known heart disease at baseline was borderline significant. In model 2, both ΔLV mass index and heart disease were significant. ΔSV2 remained significant in multivariate analysis regardless of the model used. Change in LV EF or arterial stiffness (ΔPWV) were not significantly associated with Δlog(TnT) in multivariate analysis. Baseline PWV-tertile 9.5-12.5 m/s was significant in comparison with PWV-tertile <9.5 m/s in model 2, but when baseline PWV was used as a continuous variable it was not significant in either of the two models.

*Change between baseline and 12 months*If change in TnT over 12 months was dichotomised into increase (ΔTnT>0) or decrease (ΔTnT≤0) significantly more patients in the TnT-decrease group experienced an IDH-event during follow-up (patients with ≥1 IDH-events): 13/24 (54%) in the ΔTnT≤0 group vs. 8/30 (27%) in the ΔTnT>0 group; *P*=0.04 (χ^2^-test).

*TnT-amplitude*The distribution of patients with ≥ 1 event and complete 12-months follow-up was: 15(56%) in the low amplitude group vs. 23(85%) in the high amplitude group; *P*=0.01 (χ^2^-test for admissions) and 7(26%) in the low amplitude group vs. 14(52%) in the high amplitude group; *P*=0.05 (χ^2^-test for IDH episodes).

*TnT-peak frequency*Patients without TnT-peaks tended to have fewer admissions during follow-up (patients with ≥1 admission): 29/46 (63%) in the no peak group vs. 29/35 (83%) in the ≥1 peak group; *P*=0.05 (χ^2^-test).

*Aspirin vs. non-aspirin treatment*Baseline TnT-levels tended to be higher in aspirin treated (n=37) compared to non-aspirin treated patients (n=44). Median (min-max) was: 52(14-343)ng/L (aspirin) vs. 44(44-295)ng/L (non-aspirin) corresponding to a median TnT-ratio (aspirin/non-aspirin) of 0.72(0.53-1.01); *P*=0.06. Regardless of whether change in TnT (Δ=12 months-baseline) was assessed as Δlog(TnT) or as a dichotomised outcome (increase vs. decrease) it was not significantly different when comparing aspirin treated (n=26) with non-aspirin treated (n=28). If using all available samples regardless of time in the study aspirin treated tended to have smaller median TnT-amplitude and median (min-max) TnT-amplitude was: 13(0-114)ng/L (aspirin) vs. 17(1-1611)ng/L (non-aspirin) corresponding to 27(0-214)% vs. 41(9-5197)%; *P*=0.10. Moreover, aspirin treated patients exhibited significantly fewer TnT-peaks during follow-up compared to non-aspirin treated and distribution of patients with ≥1 TnT-peak was: 11 (30%) in aspirin treated vs. 23 (52%) in the non-aspirin treated; *P*=0.04 (χ^2^-test).

**Table S1**

**Multivariate regression analysis based on baseline log(TnT)**

|  |  | **Model 1** | | | | **Model 2** | | | | **Model 3** | | | |
| --- | --- | --- | --- | --- | --- | --- | --- | --- | --- | --- | --- | --- | --- |
| Parameter |  | n | β (95% CI) | *P* | r^2^ | n | β (95% CI) | *P* | r^2^ | n | β (95% CI) | *P* | r^2^ |
| Age (years) |  | 79 | 0.01(0.00; 0.02) | 0.06 | 0.50 | 79 | 0.00(-0.01; 0.02) | 0.37 | 0.44 | 74 | 0.00(-0.01; 0.01) | 0.86 | 0.36 |
| Female gender |  | 79 | -0.19(-0.48; 0.10) | 0.19 | 0.48 | 79 | -0.36(-0.66; -0.07) | **0.02** | 0.48 | 74 | -0.18(-0.51; 0.15) | 0.27 | 0.37 |
| Cornell (S_V3_+R_aVL_) (mm) |  | 79 | 0.01(-0.01; 0.02) | 0.42 | 0.48 | 79 | 0.01(-0.01; 0.02) | 0.51 | 0.44 | 74 | 0.02(0.00; 0.04) | **0.02** | 0.40 |
| Diabetes |  |  |  |  |  | 77 | 0.38(0.06; 0.70) | **0.02** | 0.44 | 74 | 0.36(0.00; 0.72) | 0.05 | 0.39 |
| Heart disease |  |  |  |  |  | 77 | 0.15(-0.14; 0.45) | 0.31 | 0.44 | 74 | 0.31(0.00; 0.62) | **<0.05** | 0.36 |
| Charlson comorbidity index |  | 79 | 0.09(0.01; 0.18) | **0.03** | 0.47 |  |  |  |  |  |  |  |  |
| GFR (mL/min/1.73m^2^) |  |  |  |  |  |  |  |  |  | 74 | -0.06(-0.11; -0.01) | **0.03** | 0.36 |
| Urine output (L/24h) |  |  |  |  |  | 77 | -0.17(-0.37; 0.03) | 0.09 | 0.44 |  |  |  |  |
| Ultrafiltration (L) |  | 79 | 0.16(0.06; 0.27) | **0.003** | 0.47 |  |  |  |  |  |  |  |  |
| PWV-tertiles (m/s) | 9.5-12.5 | 26 | 0.42(0.09; 0.75) | **0.01** | 0.47 | 26 | 0.35(0.01; 0.69) | **0.04** | 0.44 | 26 | 0.41(0.04; 0.79) | **0.03** | 0.36 |
| (PWV <9.5 m/s = ref.) | >12.5 | 26 | 0.46(0.09; 0.83) | **0.02** |  | 26 | 0.44(0.07; 0.81) | **0.02** |  | 25 | 0.73(0.34; 1.11) | **<0.001** |  |
| LV mass index (g/m^2^) |  | 78 | 0.00(0.00; 0.00) | 0.87 | 0.47 | 78 | 0.00(0.00; 0.00) | 0.95 | 0.44 | 74 | 0.00(0.00; 0.01) | 0.29 | 0.37 |
| LV EF (%) |  | 78 | -0.02(0.03; 0.00) | **0.02** | 0.51 | 78 | -0.01(-0.03; 0.00) | 0.06 | 0.46 | 74 | -0.02(-0.03; 0.00) | **<0.05** | 0.36 |
| ΔCO (L/min) |  | 60 | -0.06(-0.23; 0.11) | 0.50 | 0.52 | 60 | -0.10(-0.25; 0.04) | 0.17 | 0.51 | 56 | -0.14(-0.30; 0.02) | 0.08 | 0.48 |
| ΔTPR (mmHg/(L/min)) |  | 59 | 0.02(-0.03; 0.07) | 0.50 | 0.52 | 59 | 0.03(-0.02; 0.08) | 0.22 | 0.50 | 55 | 0.03(-0.03; 0.09) | 0.28 | 0.45 |
| Δlog(NT-proBNP) (log(nmol/L)) | | 79 | 0.18(0.07; 0.29) | **0.002** | 0.47 | 79 | 0.24(0.12; 0.35) | **<0.001** | 0.44 | 74 | 0.19(0.07; 0.32) | **0.003** | 0.44 |
| Hematocrit (EVF) |  | 79 | -4.11(-7.18; -1.04) | **0.01** | 0.47 | 79 | -3.61(-6.90; -0.33) | **0.03** | 0.44 | 74 | -5.82(-9.43; -2.22) | **0.002** | 0.36 |

Multivariate models:

Model 1: Charlson comorbidity score, ultrafiltration volume, hematocrit (EVF), log(NT-proBNP), PWV-tertiles

Model 2: Diabetes, urine output, haematocrit (EVF), log(NT-proBNP), PWV-tertiles

Model 3: Heart disease, GFR, PWV-tertiles, EF, hematocrit (EVF)

EVF: Erythrocyte volume fraction
Abbreviations:
TnT: Troponin T; GFR: Glomerular filtration rate; PWV: Carotid-femoral pulse wave velocity; LV: Left ventricular; EF: Ejection fraction; ΔCO: Change in intradialytic cardiac output (ΔCO = CO_end_-CO_start_); ΔTPR: Change in intradialytic total peripheral resistance ((ΔTPR = TPR_end_-TPR_start_); NT-proBNP: N-terminal pro b-type natriuretic peptide; EVF: Erythrocyte volume fraction

**Table S2
Multivariate regression analysis with change in log(TnT) (Δ=12 months-baseline)**

|  |  | **Model 1** | |  |  | **Model 2** | |  |  |
| --- | --- | --- | --- | --- | --- | --- | --- | --- | --- |
| Parameter |  | n | β (95% CI) | *P* | r^2^ | n | β (95% CI) | ***P*** | **r^2^** |
| Age at baseline |  | 41 | 0.005(-0.002; 0.012) | 0.15 | 0.47 | 40 | 0.007(0.000; 0.014) | 0.05 | 0.49 |
| Heart disease |  | 41 | 0.202(-0.001; 0.407) | 0.05 | 0.44 | 40 | 0.30(0.10; 0.50) | **0.005** | 0.43 |
| Diabetes |  | 41 | -0.08(-0.28; 0.12) | 0.44 | 0.45 | 40 | -0.01(-0.22; 0.21) | 0.96 | 0.43 |
| Charlson comorbidity index |  | 41 | -0.01(-0.07; 0.05) | 0.85 | 0.44 | 40 | 0.02(-0.04; 0.08) | 0.55 | 0.43 |
| ΔGFR (mL/min/1.73m^2^) |  | 36 | -0.001(-0.07; 0.07) | 0.97 | 0.45 | 35 | 0.02(-0.05; 0.09) | 0.54 | 0.45 |
| ΔUrine output (L/24h) |  | 39 | 0.13(-0.04; 0.30) | 0.13 | 0.47 | 38 | 0.15(-0.03; 0.33) | 0.10 | 0.48 |
| ΔUltrafiltration (L) |  | 40 | -0.09(-0.20; 0.02) | 0.11 | 0.48 | 39 | -0.09(-0.21; 0.02) | 0.11 | 0.47 |
| Baseline Cornell (S_V3_+R_aVL_) (mm) |  | 41 | 0.005(-0.006; 0.015) | 0.36 | 0.45 | 40 | 0.006(-0.005; 0.017) | 0.27 | 0.44 |
| ΔCornell (S_V3_+R_aVL_) (mm) |  | 41 | -0.01(-0.02; 0.01) | 0.52 | 0.45 | 40 | -0.01(-0.03; 0.01) | 0.34 | 0.44 |
| Baseline PWV (m/s) |  | 40 | 0.003(-0.03; 0.03) | 0.86 | 0.45 | 39 | 0.01(-0.02; 0.05) | 0.34 | 0.44 |
| Baseline PWV-tertiles (m/s) | 9.5-12.5 | 13 | 0.15(-0.09; 0.39) | 0.22 | 0.48 | 12 | 0.27(0.02; 0.52) | **0.04** | 0.50 |
| (PWV <9.5 m/s = ref.) | >12.5 | 15 | 0.05(-0.18; 0.28) | 0.69 | 0.48 | 15 | 0.12(-0.11; 0.35) | 0.28 | 0.50 |
| ΔPWV (m/s) |  | 40 | -0.02(-0.07; 0.03) | 0.38 | 0.47 | 39 | -0.03(-0.08; 0.03) | 0.32 | 0.44 |
| ΔLV mass index (g/m^2^) |  |  |  |  |  | 40 | 0.004(0.001; 0.006) | **0.005** | 0.43 |
| ΔLV EF(%) |  | 40 | -0.003(-0.01; 0.01) | 0.45 | 0.45 | 40 | -0.002(-0.011; 0.007) | 0.68 | 0.43 |
| ΔSV2 (mL) |  | 41 | -0.007(-0.011; -0.003) | **0.001** | 0.44 | 40 | -0.007(-0.011; -0.003) | **0.002** | 0.43 |
| Δlog(NT-proBNP) (log(nmol/L)) |  | 41 | 0.17(0.06; 0.27) | **0.002** | 0.44 |  |  |  |  |
| ΔHematocrit (EVF) |  | 41 | -0.25(-2.44; 1.93) | 0.82 | 0.44 | 40 | -1.24(-3.46; 0.98) | 0.26 | 0.45 |

Multivariate models:
Model 1: Δlog(NT-proBNP), ΔSV(late intradialytic), heart disease
Model 2: ΔLVM-index, ΔSV(late intradialytic), heart disease

Abbreviations:
TnT: Troponin T; GFR: Glomerular filtration rate; PWV: Carotid-femoral pulse wave velocity; LV: Left ventricular; EF: Ejection fraction; SV2: Late intradialytic stroke volume 30 minutes before end of HD; NT-proBNP: N-terminal pro b-type natriuretic peptide; EVF: Erythrocyte volume fraction

**References**

1. Peters CD, Kjaergaard KD, Jensen JD, Christensen KL, Strandhave C, Tietze IN, et al. No significant effect of angiotensin II receptor blockade on intermediate cardiovascular end points in hemodialysis patients. Kidney Int. 2014;86(3):625-37.

2. Peters CD, Kjaergaard KD, Nielsen CH, Christensen KL, Bibby BM, Jensen JD, et al. Long-term effects of angiotensin II blockade with irbesartan on inflammatory markers in hemodialysis patients: A randomized double blind placebo controlled trial (SAFIR study). Hemodial Int. 2017;21(1):47-62.
